# Reviewer training to assess knowledge translation in funding applications is long overdue

**DOI:** 10.1186/s41073-017-0037-8

**Published:** 2017-08-01

**Authors:** Gayle Scarrow, Donna Angus, Bev J. Holmes

**Affiliations:** Michael Smith Foundation for Health Research, Vancouver, British Columbia Canada

**Keywords:** Peer review, Knowledge translation, Training, Health research, Funding agencies

## Abstract

**Background:**

Health research funding agencies are placing a growing focus on knowledge translation (KT) plans, also known as dissemination and implementation (D&I) plans, in grant applications to decrease the gap between what we know from research and what we do in practice, policy, and further research. Historically, review panels have focused on the scientific excellence of applications to determine which should be funded; however, relevance to societal health priorities, the facilitation of evidence-informed practice and policy, or realizing commercialization opportunities all require a different lens.

**Discussion:**

While experts in their respective fields, grant reviewers may lack the competencies to rigorously assess the KT components of applications. Funders of health research—including health charities, non-profit agencies, governments, and foundations—have an obligation to ensure that these components of funding applications are as rigorously evaluated as the scientific components. In this paper, we discuss the need for a more rigorous evaluation of knowledge translation potential by review panels and propose how this may be addressed.

**Conclusion:**

We propose that reviewer training supported in various ways including guidelines and KT expertise on review panels and modalities such as online and face-to-face training will result in the rigorous assessment of all components of funding applications, thus increasing the relevance and use of funded research evidence. An unintended but highly welcome consequence of such training could be higher quality D&I or KT plans in subsequent funding applications from trained reviewers.

## Background

Planned activities to encourage the uptake of research results by decision-makers have the potential to narrow the gap between what we know from health research evidence and what we do in practice [1–5]. The terminology designating the activities relating to moving research evidence into practice varies by discipline and geography. Variously called knowledge translation (KT, used in this paper), knowledge mobilization, evidence-informed practice, research utilization, knowledge-to-action, knowledge transfer, knowledge exchange, and dissemination and implementation (D&I) [6, 7], KT is defined by the Canadian Institutes of Health Research (CIHR) as a dynamic and iterative process that includes synthesis, dissemination, exchange and ethically sound application of knowledge to improve the health of Canadians; provide more effective health services and products; and strengthen the healthcare system [8].

As the uptake of evidence and impact for funded research gains prominence in the mandate of funding agencies [9], most now require a KT plan as a central component of funding applications. The purpose of the KT plan is to explain the relevance of the research question(s), the intended impact, and how the investigators plan to achieve that impact. The plan ideally should increase the likelihood that research evidence makes it into the hands of those who are best positioned to make use of it. Unfortunately, in the authors’ experience, the evaluation of KT plans does not tend to be done with great rigor.

Peer review as a means of assessing and assuring high-quality funded research has “often been considered the gold standard for reviewing research proposals” [10]. While there has been much debate in recent years on the effectiveness and efficiency of the peer review process (e.g., review quality, evidence of effects on relevance and accuracy of funded research, administrative burden) [11–13], efforts are being made to address these issues [10–12]. However, the debate about what constitutes good peer review and how to achieve it continues.

The authors suggest that a central reason for uneven rigor in the review of KT plans is a lack of capacity in KT. Scientific excellence is the primary criterion for peer reviewers when evaluating and funding health research. To ensure that health research funding applications are scientifically sound, by design, at least a few review panel members will be experts in each of the subject areas under consideration [11]. The problem is that KT is rarely anyone’s area of expertise. Because KT science is a relatively new area of expertise, there are few individuals who can play this role for the KT elements of grant review. This is a critical issue as funders have an obligation to ensure that the KT components of funding applications are as rigorously evaluated as the scientific components.

Steps being taken by funders to address this critical objective include:

- Providing resources for reviewers such as the Guide for Assessing Health Research Knowledge Translation (KT) Plans [14], tips for reviewers from past chairs and reviewers of KT applications [15]
- Embedding specific review criteria for KT in funding applications [16–19]
- Providing an overview of KT as part of peer review panel orientation (process followed at the Michael Smith Foundation for Health Research)
- Having KT experts on peer review panels and research users on panels as context experts to assess the merit of proposed research: its relevance to, potential uptake by, and impact on targeted stakeholders

Guidelines often tell reviewers what to look for (e.g., include knowledge users in KT plan) but not how to assess the adequacy or quality of what they are reviewing [20]. Likewise, an orientation to KT may not be sufficient to ensure that review panel members, including research users, have the knowledge and skills to adequately assess the quality of the KT plans in applications. Further, having a KT expert on the panel is problematic given that the pool of KT scientists and practitioners to draw upon is small.

In this paper, we propose that in addition to the above steps, reviewer training or education in KT, tailored to the specific requirements of the funding program, is critical to ensuring a rigorous review of KT plans and funding of excellent applications, thereby improving the likelihood that research evidence will be used to inform policy and practice. This proposal is based on a review of literature on peer reviewer training for grant review of KT, including gray literature and findings from national and international health research funders’ Web sites, interviews with key informants involved in KT across Canada, and experiential knowledge from a funder’s perspective.

## Peer review and KT—where is the evidence?

There is a scarcity of scientific evidence on the training of peer reviewers in particular in the context of funding programs [21–23]. None was found that would inform peer reviewer training in KT. What evidence is available on peer reviewer training gives us some general parameters on what might be applicable good practice for peer reviewer training in KT.

A relatively recent study on the status of biomedical grant review (USA) touches on training and guidance provided to reviewers [24]. The study found that of the 258 reviewers surveyed, only 9% (*N* = 22) had received some formal training in how to do biomedical science grant review. The instructions and guidance for external reviewers provided by funding organizations were said to be quite clear by 63% (*N* = 162), but only 16% (*N* = 42) said that these were very clear. Schroter [24] recommends that funding organizations should help reviewers do their job effectively by offering clear guidance and training as well as improved feedback and communication. Sixty-four percent (*N* = 166) said they would be interested in receiving training if funding organizations provided it free of charge.

Gelmon [25] in her article on community-based research concludes that a leader in the relevant topical field (that is, an individual whose qualifications are primarily experiential rather than academic) is a particularly important criterion for reviewing community-engaged scholarship as this type of research requires a unique expertise. The issue is how best to include these qualified individuals in the peer review process. Since few peers (academic or non-academic) are explicitly trained in peer review, she concludes that an uninitiated peer will have difficulty participating and suggests that there is a need for more formal training for academic and community peer reviewers alike.

The Michigan Institute for Clinical and Health Research (USA) proposes that research applications that include community engagement should be assessed by community research partners. They offer guidelines on how best to assess the proposals in a manner that ensures that the community’s voice is heard [19]. The guidelines cover the quality of the community-academic partnership, level of community participation, equitable distribution of budget between community and academic investigators, and relevance of the research topic to the community, as well as a project’s potential for securing external funds for a larger study.

To this end, Saunders et al. [23] note that lay community members can be trained to independently review health and medical research, and more widely involving society in funding decisions can be effectively fostered. They describe a tasked-based consumer training program developed for community review of funding applications to the Cancer Council of New South Wales (CCNSW), Australia. The program includes both didactic and practice components as well as a three-prong delivery format (oral, interaction, written) with the aim to develop the competencies needed to assess research proposals using non-scientific consumer review criteria. A separate scientific review is held and final funding decisions are made by an oversight committee using equal weighting between the scientific and community review panels. Where there is a discrepancy in scores, consensus is reached by a panel composed of both trained community members and researchers.

Ruppertsberg et al. [16] have developed audit criteria for researchers and their organizations to assess the KT plans of the researchers’ health funding proposals for the purpose of identifying areas for improvement. While not aimed specifically at peer reviewers, this exploratory work may prove of some relevance for funders developing training material for peer reviewers to assess the quality of the KT components of funding applications.

A 2009 review of peer review studies by the RAND Corporation (USA) included a list of possible modifications to peer review. To improve effectiveness of the peer review process and strengthen reliability, more effective training for peer reviewers was found to have insufficient evidence to draw conclusions on the advantages or disadvantages of such an approach [16]. However, the review does not define effective training.

While there is a paucity of scholarly evidence related to KT reviewer training in the literature, a review of funders’ websites found a range of activities in support of peer review and training. As noted above, the Cancer Council New South Wales (CCNSW), Australia [26], provides peer review training for community members in research and research review as well as an assessment tool that includes criteria reflecting community values such as real world applicability of the research, its potential for positive impact, the availability of research findings to those who could benefit from it, whether consumers were involved in the research, and the length of time to availability of research evidence in practice [27].

The Canadian Institutes for Health Research (CIHR) (Canada) peer review has undergone a reform under the oversight of its College of Reviewers [28] whose role is to enhance CIHR’s current peer review system to reduce the burden and challenges associated with it that are felt by peer reviewers and applicants. Objectives specific to reviewer expertise and training include the following: (1) systematizing reviewer recruitment to identify and mobilize the appropriate expertise for all funding applications and (2) developing customized learning and mentoring programs to provide reviewers with the knowledge and resources necessary to conduct consistent and fair reviews. CIHR has developed compulsory learning modules (available on line) on grant review. In the future, these learning modules will include a specialized module on knowledge translation.

The Patient-Centered Outcomes Research Institute (USA) trains reviewers on the merit review process and the merit-review criteria through a series of webinars, online training, online manuals, and a methodology 101 for research-user panel members [29]. These criteria differ from those of most scientific reviews as in addition to scientific rigor, they include patient-centeredness, the engagement of patients and stakeholders in the conduct of the research, and the likelihood that the research could change patient or clinician practices.

## Peer review and KT—what do the key informants suggest?

The following questions were explored in interviews with key informants (nine in total), who were KT scientists and KT professionals at health research, health research funding, and health provider agencies across Canada: (1) do peer review panels require an in-depth understanding of KT in order to properly review grant applications, (2) should KT expertise on a peer review panel be provided through experts on the panel or training the panel in how to review KT or both, and (3) if there is training, what should the training look like. The key informants said that the answers to those questions were dependent on the following factors:

### The importance of KT to the kind of grant being reviewed

If KT is truly important to the funding program, then review of the KT components of the grant proposals should have clear criteria, training for the review panel, expert oversight where appropriate (through internal or external expert opinion or having experts on the panel), and reviewer buy-in (clear expectations and role definition). If KT is less important, then the amount of effort and emphasis can be adjusted accordingly.

### The type of KT being reviewed

End-of-grant KT, also termed dissemination, will require a different level of oversight from KT that is integrated throughout the research program. There would be, for example, different KT expertise required for an end-of-grant KT plan for a basic biomedical science proposal versus a KT plan for a study of patient outcomes and practice variation following implementation of an intervention co-designed with research-user partners. As well, the review of KT plans for longer term and more complex research should not be done solely at the application stage but revised and reassessed as the research progresses.

### The review panel’s level of knowledge and experience in KT, including the Chair

Training requirements should consider whether reviewers have served on panels before and have already gone through orientation and training or are reviewers with KT expertise or experience versus new recruits with little or no background in KT.

### The resources the organization has to do training

What is optimal and what is feasible may not be the same thing. Resources are becoming available for training [7, 19, 24] so there should be no need to reinvent every wheel but rather to repurpose existing resources, such as guidelines, to suit an individual funder’s needs.

In summary, the key informants felt that reviewers do need a solid understanding of what good KT plans should contain and guidelines are not sufficient for this purpose. Training should therefore be required. Training should be delivered just in time and should recognize existing levels of expertise. Having an expert on every panel is an alternative but is not be feasible given the small pool of available experts.

## Discussion

There is no evidence (scientific, from the key informants interviews, or experiential knowledge) that points to a single best practice for training reviewers in KT. Nevertheless, there is some guidance from learning theory and adult education principles. We know, for example, that people learn in different ways, and therefore, it seems reasonable to apply a combination of learning approaches that take into account the level of prior knowledge, ability, and interests of a particular set of students [30]. Knowles’ adult learning principles can also inform development of training opportunities; that is, adults are most interested in subjects of immediate relevance and impact to their jobs and lives; their past experiences provide the basis for the learning activities; adults need to be involved in the planning and evaluation of their instruction; and adult learning is problem-centered rather than content-oriented [31]. When considering KT training for reviewers for example, opportunities should be varied, appropriate to the context, build on their prior knowledge and experience, involve reviewers in their development and evaluation, and include multiple learning styles. For example, training could include online options, printed materials and resources in the form of modules, and/or face-to-face training such as vignettes and mock reviews or reviewing actual applications to show what good KT (contains *x*, *y*, and *z*) and not-so-good KT looks like.

We know from experience and from the literature search that it is the practice of funding agencies to have guidelines in place for peer review of various aspects of applications, including KT. We would propose that KT training for reviewers be supported with funding program guidelines that clearly articulate the funder’s expectation of excellence in KT planning. Funders should build a set of application and matching review criteria for each kind of KT (end-of-grant and integrated throughout the research process), with reviewer guidelines that have enough detail to support an assessment of the adequacy of an application’s KT components based on the type of research, the stage it is at, the expected findings, and the target audience(s). The guidelines should help the reviewer assess what difference this research will make; how the knowledge sharing activity will benefit research users and or/decision makers; what is the potential to inform decision-making in clinical practice, community programming, and government or agency policy; and how the project activities will engage research users in co-developing, sharing, and disseminating this knowledge.

Including research users on review panels not only serves the purpose of ensuring relevance and feasibility which are considered in funding decisions [32] but also serves to educate other panel members. The value of including research users on review panels where the type of research requires a unique expertise has been identified in community-based research [25] and research within Indigenous communities [33].

The results of the key informant interviews and experiential knowledge tell us that it is sometimes difficult to get researchers and peer review panel members to understand this requirement on engagement. The use of examples and frequently asked questions (FAQs) were suggested by key informants. As well, definitions on engagement must be clear in guidelines for reviewers and researchers alike; for example, “participatory approach” may mean something different to researchers than research users or between different user groups (e.g., clinicians versus patients).

A KT expert is more appropriate on grants that have a substantive or more involved KT component, although it was suggested by the key informants that having an expert to review dissemination plans may still have value. For more involved KT, it may be important to have more than one kind of KT expert—commercialization for example or implementation science. Having KT experts on panels also provides learning opportunities for the panel through the discussion of the KT portions of applications during the review. The degree and type of KT learning for reviewers through panel discussions relates to the degree and type of KT expertise which is a function of the nature of the research under review.

Guidelines for assessment of KT in funding applications can also be used as a basis to train reviewers. One example is the *Guide for Assessing Health Research Knowledge Translation* (*KT*) *Plans* (2007) [18] that was developed as part of a research study by investigators at McMaster University, Canada. It has become a key source document for the development of guidelines for researchers at CIHR, Canada’s national health research funding agency, as well as other agencies. The guide had two purposes: to provide reviewers with conceptual tools to do assessments of the KT components of plans and to provide applicants with information about how their plans will be assessed. The guide also informed the development of applicant guidelines.

There is the danger that seasoned reviewers will be unwilling to participate in training on something they feel they already know so training needs to be calibrated to the knowledge level of the reviewers. Training is also time-consuming so it was further suggested by some of the key informants that training could be embedded in orientation as part of a pre-meeting. It was also suggested that the panel could score the applications before coming to the meeting and then calibrate the KT as part of the understanding that at the review meeting, the scores can be changed. One key informant who has served as a panel chair on numerous occasions conducts a calibration exercise for KT components of applications at the beginning of all panel review meetings—in effect, a form of KT training.

**Fig. 1:**
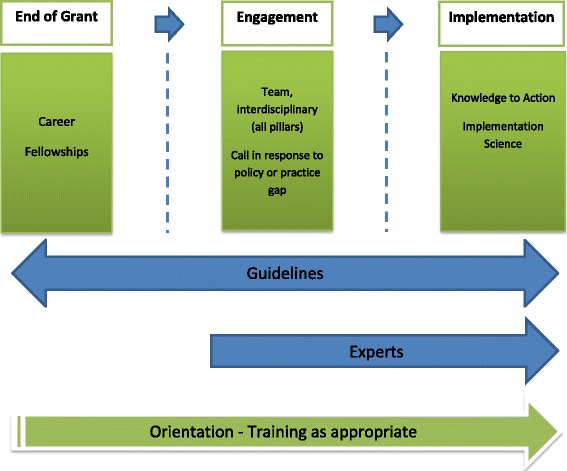
Considerations in peer review of KT in grant applications

Figure 1 depicts the general relationship between various programs, type of KT and requirements for guidelines, need for KT experts, and need to train reviewers. The dotted line between the types of grant programs represents the blurring of the distinction between types of KT for various types of funding programs. Table 1 proposes a way of assessing the appropriate mix of panel composition, orientation, and training.

**Table 1:** Variable approach to knowledge translation training for programs

Program	Orientation	Guidelines	Training	Experts
Early career salary	✔	✔		
Post-doctoral fellowship	✔	✔	✔(Unless KT expert on panel)	✔If a KT Science application
KT broker	✔	✔		✔
Researcher—research user partnerships, e.g., research-to-action, interdisciplinary, and implementation science	✔	✔	✔	✔

## Conclusion

With the increasing emphasis on realizing impact from research, funders have an obligation to ensure that the KT components of funding applications are as rigorously evaluated as the scientific components. Hearteningly, some funders are beginning to address the issue of peer review of KT through increased oversight, inclusion of appropriate experts and research users on review panels, and guidelines with clear expectations for the KT components of applications and their review. As virtually all research grants, particularly those in the health sector, now require some form of KT planning, all reviewers will need some degree of understanding of these elements in order to appropriately review them. Based on a review of the literature, funding agency Web sites, interviews with key informants, and experiential knowledge gained within health research funding agencies, we conclude that these actions may be insufficient on their own and consideration should be given to KT training for reviewers.

What has also emerged from this review is that there is little or no evidence to support the efficacy of KT training for grant peer reviewers nor that can inform best practices in its design and delivery. In making recommendations, we have relied on extrapolated evidence as well as expert and experiential knowledge. There is a pressing need for research in this area.

A great deal is at stake in peer review and with that comes an obligation to ensure that peer reviewers have all the support they require to fulfill their responsibilities, particularly in the review of KT plans. By providing reviewers with the competencies they require, an important step will have been taken towards ensuring that funded research is not only excellent science but also relevant and useful to society.

## Abbreviations

CIHR: Canadian Institutes of Health Research
